# Supplementary material for: Formation and Long-Term Culture of hiPSC-Derived Sensory Nerve Organoids Using Microfluidic Devices
Source: Bioengineering (Basel). 2024 Aug 5;11(8):794. doi: 10.3390/bioengineering11080794 (PMC11352057; doi:10.3390/bioengineering11080794)
Supplement: Supplementary file 1 [file bioengineering-11-00794-s001.zip › SupplementaryMaterials_SNO.pdf]

bioengineering

Supplementary Materials

Formation and Long-Term Culture of hiPSC-Derived Sensory Nerve Organoids Using Microfluidic Devices

Takuma Ogawa [1], Souichi Yamada [2], Shuetsu Fukushi [2], Yuya Imai [1], Jiro Kawada [3], Kazutaka Ikeda [4,5], Seii Ohka [4] and Shohei Kaneda [1,*]

1 Mechanical Engineering Program, Graduate School of Engineering, Kogakuin University, 1-24-2 Nishishinjuku, Shinjuku-ku, Tokyo 163-8677, Japan;
2 Department of Virology I, National Institute of Infectious Diseases, 1-23-1 Toyama, Shinjuku-ku, Tokyo 162-8640, Japan;
3 Jiksak Bioengineering, Inc., 3-25-16 Tonomachi, Kawasaki-ku, Kawasaki, Kanagawa 210-0821, Japan;
4 Addictive Substance Project, Tokyo Metropolitan Institute of Medical Science, 2-1-6 Kamikitazawa, Setagaya-ku, Tokyo 156-8506, Japan; ohka-si@igakuken.or.jp (S.O.)
5 Department of Neuropsychopharmacology, National Institute of Mental Health, National Center of Neurology and Psychiatry, 4-1-1 Ogawahigashi-cho, Kodaira, Tokyo 187-8553, Japan
* Correspondence: kaneda@cc.kogakuin.ac.jp

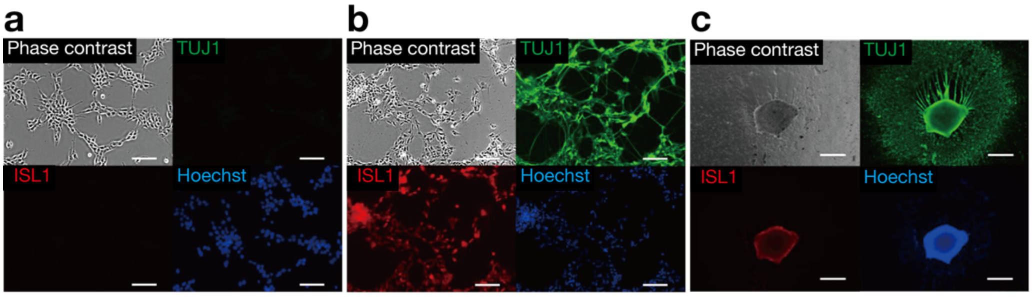

Figure S1. Characterization of iPSCs, induced sensory neurons, and neurospheres using fluorescent immunostaining for TUJ1 as a neuronal marker and ISL1 as a sensory neuron marker. (a) iPSCs before induction of sensory neurons. (b) Induced sensory neurons. (c) Neurosphere composed of induced sensory neurons, seeded in a dish and cultured for 12 days. Scale bars: 100 μm in (a) and (b), 500 μm in (c).

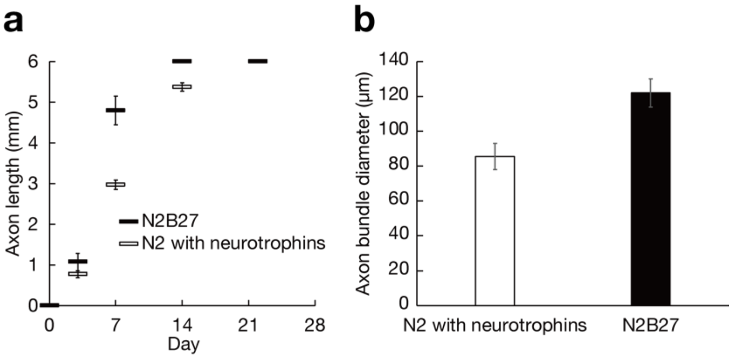

Figure S2. Axon length and axon bundle diameter of sensory nerve organoids cultured using medium with and without neurotrophins. (a) Axon length. (b) Bundle diameter. $n = 4$.
